# Functional estrogen receptor signaling pathway activity in high-grade serous ovarian carcinoma as compared to estrogen receptor protein expression by immunohistochemistry

**DOI:** 10.1007/s13402-021-00600-5

**Published:** 2021-03-16

**Authors:** Phyllis van der Ploeg, Laura A. M. van Lieshout, Anja van de Stolpe, Steven L. Bosch, Marjolein H. F. M. Lentjes-Beer, Ruud L. M. Bekkers, Jurgen M. J. Piek

**Affiliations:** 1 Department of Obstetrics and Gynecology, Catharina Hospital, Michelangelolaan 2, 5623 EJ Eindhoven, The Netherlands; 2 GROW School for Oncology and Developmental Biology, Maastricht University, Maastricht, The Netherlands; 3 Radboud Institute for Health Sciences, Department of Obstetrics and Gynecology, Radboud University Nijmegen Medical Center, Nijmegen, The Netherlands; 4 Molecular Pathway Dx, Philips, Eindhoven, The Netherlands; 5 Laboratory for Pathology and Medical Microbiology (Stichting PAMM), Eindhoven, The Netherlands; 6 Laboratory for Pathology, Jeroen Bosch Hospital, ‘s-Hertogenbosch, The Netherlands

**Keywords:** Estrogen receptor, Immunohistochemistry, Signaling pathway activity, High-grade serous ovarian carcinoma

## Abstract

**Purpose:**

Anti-estrogen therapy may be used as a palliative treatment option in high-grade serous ovarian carcinomas (HGSC). However, clinical implementation is limited as the use of estrogen receptor (ER) protein expression by immunohistochemistry remains insufficient in predicting therapy response. To determine the accuracy of ER protein expression as a marker for ER signaling pathway activity, we aimed to correlate ER protein expression to functional ER signaling pathway activity in HGSC.

**Methods:**

Immunohistochemical ER protein expression was visually scored using total percentages of stained tumor cells and histoscores. Subsequently, mRNA was extracted, and RT-qPCR analysis was performed. Functional ER pathway activity was assessed by a computational Bayesian model inferring ER signaling pathway activity from mRNA levels of ER-specific target genes.

**Results:**

Our analysis of 29 HGSCs shows that neither total percentage of ER protein expression, nor ER histoscores are significantly correlated to ER signaling pathway activity (respectively, *p* = 0.473 and *p* = 0.606). Classification of HGSC into three groups based on ER histoscores 0–100 (n = 6), 101–200 (n = 15) and 201–300 (n = 8) resulted in comparable mean ER signaling pathway activity among the groups (*p* = 0.356). Several samples in the higher ER histoscore groups had low ER signaling pathway activity, indicating that nuclear ER protein expression is not sufficient to describe transcriptional ER activation.

**Conclusion:**

Positive immunohistochemical ER staining is not always indicative of an active ER signaling pathway and is, therefore, a poor predictor of anti-estrogen response. Further research is needed to prove the predictive value of ER signaling pathway activity regarding anti-estrogen sensitivity in HGSC patients.

**Supplementary Information:**

The online version contains supplementary material available at 10.1007/s13402-021-00600-5.

## Introduction

Anti-estrogen targeted therapy has been studied extensively in recurrent and metastatic ovarian carcinoma during the past decades [1]. Although anti-estrogen therapy generally has an excellent tolerability, the best therapeutic response is often disease stabilization, yielding a clinical benefit in 27–65% of high-grade serous ovarian carcinomas (HGSCs) and 64–71% of low-grade serous ovarian carcinomas (LGSCs) [2–4]. The implementation of anti-estrogen therapy in the clinical setting is limited as reliable predictive biomarkers for the identification of sensitive ovarian carcinomas are lacking.

The most studied marker for anti-estrogen therapy response is nuclear estrogen receptor-alpha (ER) protein expression by immunohistochemistry [5]. The presence of nuclear ER varies considerably between histological subtypes, as 71% of LGSC and 60% of HGSC are indicated ER positive compared to 14% of clear cell carcinomas [6]. Currently, positive ER staining is considered indicative of an active ER signaling pathway, and endocrine sensitivity is suggested to correspond with ER status. However, evidence for a predictive correlation remains weak as multiple studies failed to discover a significant relation between increasing ER protein expression and improved response to anti-estrogen therapy [4, 7–11]. Therefore, we question the accuracy of ER protein expression as a marker for ER signaling pathway activity in ovarian carcinomas.

In the absence of an activating mutation, the substrate estradiol is required to activate the ER and initiate transcription of ER target genes [12]. While immunohistochemical staining of nuclear ER demonstrates the presence of the receptor, it might be insufficient to indicate functional ER activation. In order to assess transcriptional ER signaling pathway activity, an ER pathway activity model based on measurements of mRNA levels of ER-specific target genes has been developed [13, 14]. Previous use of the ER pathway activity model in breast cancer patients produced functional ER signaling pathway activity scores with better predictive value regarding anti-estrogen therapy response than ER protein expression [13, 15, 16].

As the ER signaling pathway is considered a potential target for therapy in a subset of HGSC, the most common histological subtype of ovarian cancer, reliable markers to identify this anti-estrogen sensitive subset are needed. In this study we investigate whether ER protein expression correlates to functional ER signaling pathway activity in advanced stage HGSC in the search for an alternative predictive biomarker for anti-estrogen therapy response.

## Materials and methods

### Patient population and data collection

We retrospectively selected patients diagnosed with advanced stage HGSC in the Catharina Hospital Eindhoven, The Netherlands. To prevent any interference of cytotoxic or anti-estrogen therapy with ER signaling pathway activity measurements, patients were excluded if (1) formalin-fixed paraffin-embedded (FFPE) tumor samples were obtained after the start of chemotherapy, (2) patients had a medical history of any other malignancy prior to HGSC diagnosis, with the exception of basal cell skin carcinoma and (3) medical records stated recent use of oral contraceptives or hormone replacement therapy. We retrieved the following data from medical records: age and menopausal status at diagnosis, FIGO stage and tumor origin. In case medical records lacked information on menopausal status and age at diagnosis was insufficient to confirm postmenopausal status, endometrial sections were reviewed to determine menopausal status [17]. Tumor histology was confirmed by an expert gynecological pathologist (MHFML-B) and areas containing at least 30% tumor cell nuclei were annotated for further analysis.

### ER protein expression by immunohistochemistry

FFPE sections of 4 μm were cut with a microtome and dried at 80°C in a convection oven for 20 min. Fully automated immunohistochemical staining for the detection of ER-alpha was performed on a BOND III stainer (Leica Biosystems, Germany). Slides were incubated with rabbit monoclonal antibody (SP1) (1:60, Thermo Scientific, USA) for 30 min at 20°C after heat-induced epitope retrieval with ER-2 (ethylenediaminetetraacetic acid-based buffer, pH 9, Leica Biosystems, Germany) for 30 min at maximum 100°C. Detection of the primary antibody was performed using Bond Polymer Refine Detection (Leica Biosystems, Germany), including two incubation steps of 8 min at 20°C. Positive cells were visualized after 10 min incubation with 3,3-diaminobenzidine/H_2_O_2_ (Leica Biosystems, Germany) at room temperature.

### ER protein expression scoring methods

Two expert gynecological pathologists (SLB and MHFML-B) independently determined ER protein expression in the annotated tumor areas. The pathologists were blinded for each other’s assessment and the results of the ER pathway activity model. ER protein expression was visually scored according to two methods. First, ER protein expression was estimated by the total percentage of positive stained tumor cell nuclei. Second, staining intensity was categorized in percentages of tumor cells with negative, weak, moderate or strong staining. Finally, ER histoscores were calculated using a weighted method from the sum of (1 x % weak cells) + (2 x % moderate cells) + (3 x % strong cells), deriving histoscores between 0 and 300 [18]. Mean scores for both ER protein expression scoring methods were calculated and used for further analysis.

### mRNA extraction and RT-qPCR analysis of ER pathway-specific target genes

Consecutive FFPE sections of 5 μm with identical annotated tumor areas were manually scraped for the collection of tumor tissue. Depending on total annotated tumor area, multiple sections were macro-dissected resulting in at least 20 mm^2^ tumor surface. Total mRNA was extracted according to the manufacturer’s protocol (VERSANT® Tissue Preparation Reagents kit, Siemens, Germany). mRNA concentrations were measured using a Qubit® RNA HS Assay Kit and Qubit® Fluorometer (Invitrogen, Thermo Fisher Scientific, USA). Real-time quantitative reverse transcription polymerase chain reaction (RT-qPCR) analysis was performed using a SuperScript™ III Platinum™ One-Step qRT-PCR kit (Invitrogen, Thermo Fisher Scientific, USA). OncoSignal PCR plates (Philips MPDX, The Netherlands) were filled with one nanogram mRNA per well and processed using a CFX96 Real-Time PCR Detection System (BioRad, USA). Sufficient mRNA input was confirmed by an internal quality control of reference genes.

### ER pathway activity model

Functional ER signaling pathway activity was assessed using an OncoSignal pathway assay (OncoSignal, Philips MPDx, The Netherlands), which is based on mRNA expression levels of ER-specific target genes and has been described in detail previously [13, 14]. An extensive literature search yielded 27 ER pathway-specific target genes for the Affymetrix ER pathway activity model [13]. The model has been developed and validated on Affymetrix expression microarray data [19]. For the use on FFPE material, the model was adapted based on a selection of the most informative ER target genes as has been described before [15, 20]. The ER pathway activity model consists of 3 nodes corresponding to (i) the ER transcription complex, (ii) ER target genes and (iii) measured probeset expression levels. The activity of the ER transcription complex (i) is inferred from the expression of ER target genes (ii and iii) by a computational Bayesian network. The ER pathway activity model generates functional scores defined on a normalized scale from 0 to 100, where 0 corresponds to the lowest odds for an active ER signaling pathway and 100 corresponds to the highest odds for an active ER signaling pathway. However, the biological range of ER pathway activity scores will differ between various tissue types. All samples were analyzed blinded for ER protein expression levels.

### Statistical analysis

Intra-class correlation coefficients were determined with a two-way mixed model to test the overall concordance between total percentage ER protein expression and ER histoscore assessments of both pathologists. Differences in mean ER signaling pathway activity between ER histoscore groups were tested using a Kruskal-Wallis test. Spearman’s rank-order correlation coefficient was used to assess correlations. Statistical testing results were considered significant if the *p* value was below 0.05. All statistical analyses were conducted using SPSS (IBM SPSS Statistics, version 26, RRID:SCR_019096) and RStudio (RStudio, Inc. version 1.1.463, RRID:SCR_000432) was used for data visualization.

## Results and discussion

In this study we included 29 patients diagnosed with advanced stage HGSC with a median age at diagnosis of 63 years (range 31–85 years). Four patients were premenopausal at the time of diagnosis, one patient was considered perimenopausal and 23 patients were postmenopausal. For one patient we were unable to determine menopausal status as endometrial sections were unavailable for revision and age at diagnosis was insufficient to confirm postmenopausal status. Patients were diagnosed with FIGO stage IIIC (72%) or IV (28%) disease and tumor origin was defined as ovarian (83%), Fallopian tube (7%) and extra-ovarian (10%) (Table 1).

**Table 1:** Clinicopathological characteristics of patients diagnosed with high-grade serous ovarian carcinoma

	*n* = 29 (%)
Age at diagnosis	
Median (range)	63 (31–85)
Menopausal status	
Premenopausal	4 (14)
Perimenopausal	1 (3)
Postmenopausal	23 (79)
Unknown	1 (3)
Tumor origin	
Ovarian	24 (83)
Fallopian tube	2 (7)
Extra-ovarian	3 (10)
FIGO stage	
IIIC	21 (72)
IV	8 (28)

**Fig. 1:**
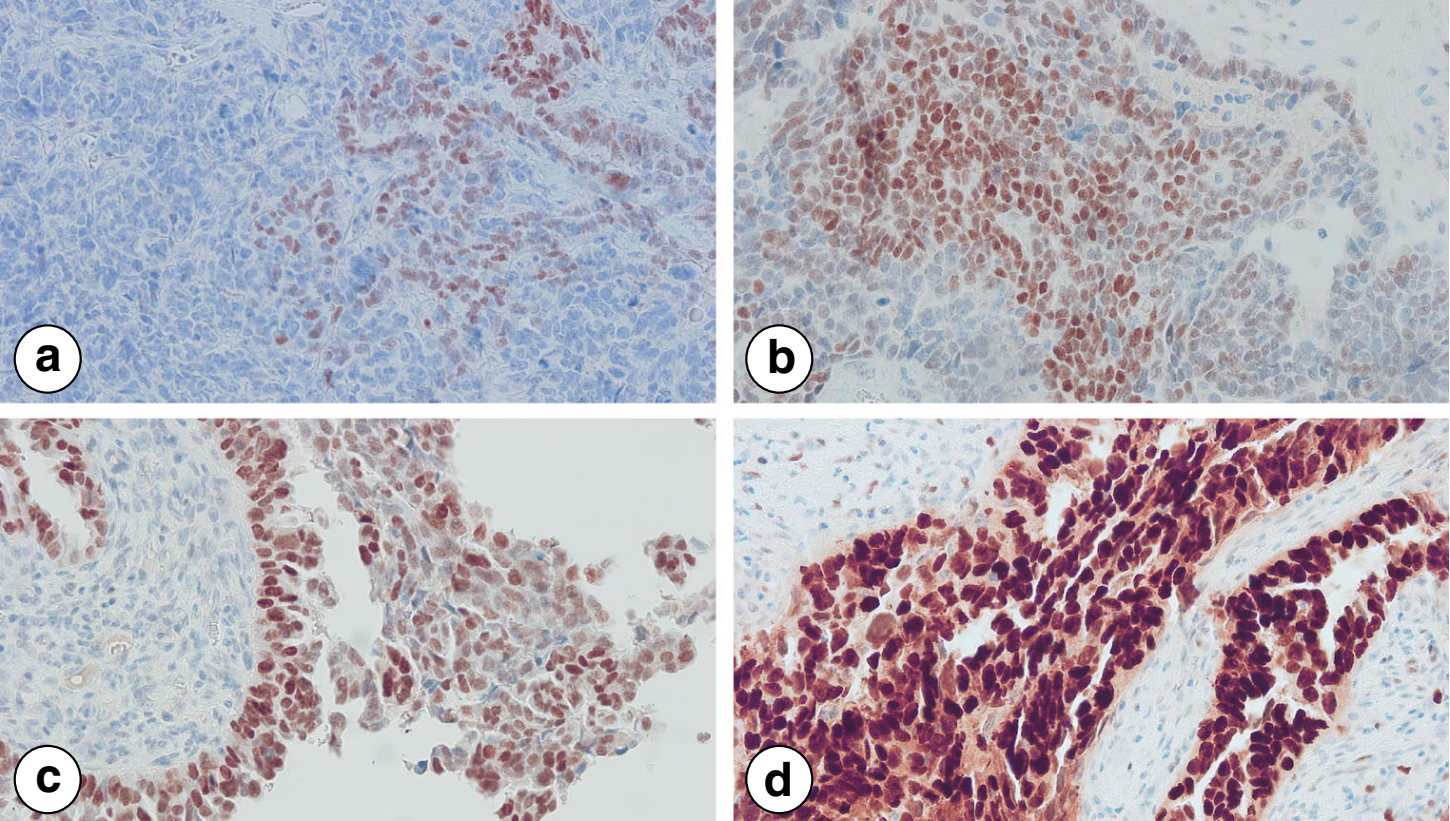
Estrogen receptor (ER) nuclear protein expression assessed by immunohistochemistry in high-grade serous ovarian carcinoma samples. Representative images (20x magnification) illustrate the following ER staining intensities: **a** predominant negative (1% ER expression and ER histoscore of 1), **b** predominant weak (85% ER expression and ER histoscore of 138), **c** predominant moderate (95% ER expression and ER histoscore of 190) and **d** predominant strong (100% ER expression and ER histoscore of 278)

Both gynecological pathologists assessed ER protein expression according to two methods in 29 HGSC samples (Supplementary Table 1). Figure 1 shows representative images of immunohistochemical ER staining intensities with corresponding total percentage ER protein expression and ER histoscores. Interobserver agreement for both assessments was excellent with an intra-class correlation coefficient based on absolute agreement of 0.968 (95% Confidence Interval (CI) 0.927–0.985) for total percentage ER protein expression and 0.919 (95% CI 0.526–0.974) for ER histoscores. The majority of the HGSC samples showed positive ER expression with a mean total percentage ER protein expression of 74% (standard deviation (SD) 36%) and a mean ER histoscore of 161 (SD 89) (Table 2). We classified the HGSC samples in three groups based on ER histoscores 0–100 (n = 6), 101–200 (n = 15) and 201–300 (n = 8).

**Table 2:** Descriptive statistics of total percentage estrogen receptor (ER) protein expression, ER histoscores and ER signaling pathway activity in high-grade serous ovarian carcinoma samples

Method	n	Mean	Standard deviation	Median	Range
Total percentage ER protein expression	29	74%	36%	90%	0–100%
ER histoscores	29	161	89	175	0–278
ER signaling pathway activity scores	29	12.09	6.40	11.30	0.22–27.94

**Fig. 2:**
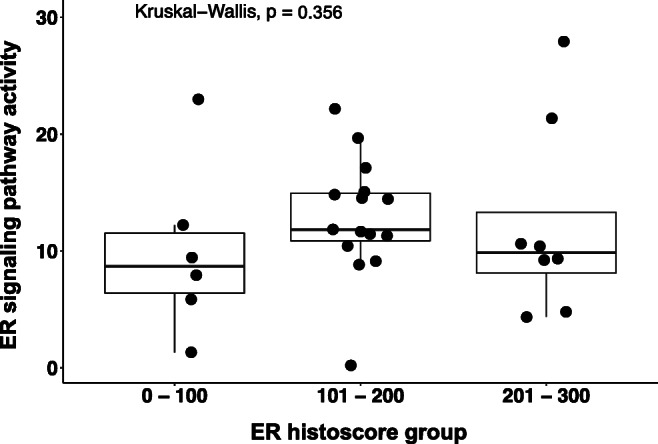
Estrogen receptor (ER) signaling pathway activity per ER histoscore group determined in high-grade serous ovarian carcinoma samples (*p* = 0.356)

Functional ER signaling pathway activity was determined in the same 29 HGSC samples. The mean ER signaling pathway activity was 12.09 (SD 6.40) (Table 2). When grouped according to ER histoscores (0–100, 101–200 and 201–300), we measured mean ER signaling pathway activities of 9.97 (SD 7.36), 12.85 (SD 5.13) and 12.26 (SD 8.20), respectively (Fig. 2). We observed a wide variation in ER signaling pathway activity within the three ER histoscore groups. Although the lower ER histoscore group showed the lowest mean ER signaling pathway activity, this was not statistically different from the mean ER signaling pathway activity measured in the higher ER histoscore groups (Fig. 2) (*p* = 0.356). In these higher ER histoscore groups, we observed several HGSC samples with low ER signaling pathway activity, indicating that presence of nuclear ER is required, but not sufficient to prove a transcriptionally active ER signaling pathway.

**Fig. 3:**
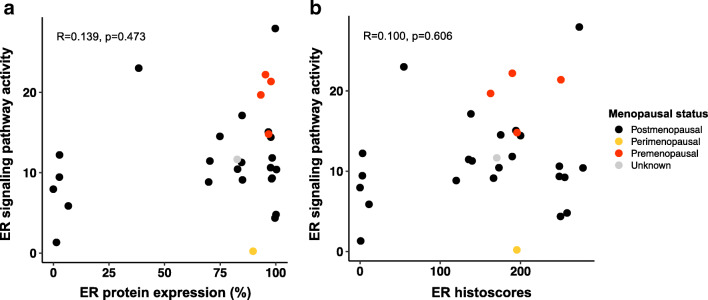
Relation between estrogen receptor (ER) protein expression and ER signaling pathway activity in high-grade serous ovarian carcinoma samples. ER protein expression defined as: **a** total percentage positive stained tumor cell nuclei (R = 0.139, *p* = 0.473) and **b** ER histoscores (R = 0.100, *p* = 0.606)

Next, we investigated the correlation between ER protein expression and functional ER signaling pathway activity. For both ER scoring methods, we observed no statistically significant correlation with ER signaling pathway activity (total percentage ER protein expression (R = 0.139,* p *= 0.473) and ER histoscores (R = 0.100, *p* = 0.606) (Fig. 3)). These results indicate that ER protein expression is not equivalent to transcriptional ER signaling pathway activity in HGSC and may explain the insufficiency of ER protein expression as a predictive marker for anti-estrogen therapy response.

In premenopausal HGSC (n = 4) we measured significant higher ER signaling pathway activity compared to postmenopausal HGSC (n = 23) (mean ER signaling pathway activity, respectively, 19.52 and 11.33, *p* = 0.014). The association between ER signaling pathway activity and menopausal status indicates that the biological availability of estradiol in premenopausal women may affect the tumor’s sensitivity to hormones. Since in premenopausal women estradiol is constantly produced by the ovaries, the tumor’s signaling pathway activity could become dependent on the paracrine and endocrine availability of estradiol. With increasing age, the depletion of ovarian follicles causes a steady decline in estradiol production by the ovary [21]. After menopause, when the estradiol production of the ovary has ceased, the formation of estradiol depends on the availability of androgens and estrogen precursors [22]. In postmenopausal women, tumor cells are thought to produce estradiol by the aromatase and sulfatase pathways [23]. In the aromatase pathway, estradiol is formed by intracellular conversion of androgens, which is mediated by the enzyme aromatase. In the sulfatase pathway, estradiol is mainly produced from the inactive precursor estrone sulfate. Therefore, in postmenopausal women, high ER signaling pathway activity in HGSC is likely to be caused by local estradiol production of the tumor itself (autocrine production) or, alternatively, by extragonadal production in liver, brain or adipose tissue [22, 23]. Again, these results indicate that presence of the ER is a prerequisite, but that transcriptional ER signaling pathway activity depends on the availability of ligand. We hypothesize that, despite positive ER protein expression, only high ER signaling pathway activity represents functionally active ER signaling in HGSC and that, therefore, only these HGSC patients are likely to benefit from anti-estrogen targeted therapy.

Our findings are supported by a study on ER signaling pathway activity in 130 ER positive breast cancer patients using the ER pathway activity model [16]. In this cohort, ER protein expression was also not significantly correlated with ER signaling pathway activity (*p* = 0.400). In addition, the authors reported no correlation between *ESR1* gene product (ER-alpha) levels, and ER signaling pathway activity (*p* = 0.510). Others studied ER signaling pathway activity in a cohort mainly consisting of endometrial cancer patients (n = 83) using the ER pathway activity model [20]. Here, significantly lower ER signaling pathway activity was observed in the group with 0–10% ER protein expression compared to the group with 51–100% ER protein expression (*p* < 0.001). In line with our observations, a wide variation in ER signaling pathway activity was detected in the higher ER protein expression groups (11–50% and 51–100%), indicating that positive ER protein expression in endometrial cancer also did not automatically imply transcriptional activation of the ER signaling pathway.

Besides the limited number of patients included, our study lacks anti-estrogen response data, as the included HGSC patients did not receive anti-estrogen therapy. Therefore, we were unable to study the predictive value of the ER pathway activity model regarding anti-estrogen response. However, in multiple cohort studies the model was able to select subsets of ER positive breast cancer patients with an active ER signaling pathway and a significantly better response to anti-estrogen targeted therapy [13, 15, 16]. Our group is currently investigating whether ER signaling pathway activity is associated with anti-estrogen therapy response in low-grade ovarian carcinomas. The search for a predictive marker remains warranted as clinical studies report subsets of ovarian carcinoma patients who benefit from anti-estrogens with minimal side effects. However, in order to implement anti-estrogen targeting as an effective treatment strategy a more reliable marker is required, as ER protein expression alone remains insufficient in predicting anti-estrogen sensitivity.

Taken together, our data indicate that ER protein expression as detected by immunohistochemistry in HGSC not always translates into active ER signaling pathway activity based on mRNA levels of ER-specific target genes. Further investigation is necessary to confirm ER signaling pathway activity as a predictive marker for response to anti-estrogen therapy.

## Supplementary Information

ESM 1 (DOCX 23 kb)

## Data Availability

Additional data underlying this article are available from the corresponding author on reasonable request.
