# Supplementary material for: Functional estrogen receptor signaling pathway activity in high-grade serous ovarian carcinoma as compared to estrogen receptor protein expression by immunohistochemistry
Source: Cell Oncol (Dordr). 2021 Mar 16;44(4):951–7. doi: 10.1007/s13402-021-00600-5 (PMC8338831; doi:10.1007/s13402-021-00600-5)
Supplement: Supplementary file 1 — (DOCX 23 kb) [file 13402_2021_600_MOESM1_ESM.docx]

**Functional estrogen receptor signaling pathway activity in high-grade serous ovarian carcinoma as compared to estrogen receptor protein expression by immunohistochemistry**

Journal: Cellular Oncology

Phyllis van der Ploeg^1,2*^, Laura A.M. van Lieshout^1,3^, Anja van de Stolpe^4^, Steven L. Bosch^5^, Marjolein H.F.M. Lentjes-Beer^6^, Ruud L.M. Bekkers^1,2^, Jurgen M.J. Piek^1^

1. Department of Obstetrics and Gynecology, Catharina Hospital, Eindhoven, The Netherlands
2. GROW School for Oncology and Developmental Biology, Maastricht University, Maastricht, The Netherlands
3. Radboud Institute for Health Sciences, Department of Obstetrics and Gynecology, Radboud University Nijmegen Medical Center, Nijmegen, The Netherlands
4. Molecular Pathway Dx, Philips, Eindhoven, The Netherlands
5. Laboratory for Pathology and Medical Microbiology (Stichting PAMM), Eindhoven, The Netherlands
6. Laboratory for Pathology, Jeroen Bosch Hospital, ‘s-Hertogenbosch, The Netherlands

*Correspondence address: Catharina Hospital, Department of Obstetrics and Gynecology, Michelangelolaan 2, 5623 EJ Eindhoven, The Netherlands. E-mail: phyllis.vd.ploeg@catharinaziekenhuis.nl

**Table S1** Specification of high-grade serous ovarian carcinoma samples

| **Study number** | **Age at diagnosis** | **Menopausal status** | **Tumor cell nuclei in annotated area (%)** | **ER positive stained tumor cell nuclei (%)^*^** | **ER histoscore^*^** | **ER signaling pathway activity** |
| --- | --- | --- | --- | --- | --- | --- |
| 01219545 | 79 | Postmenopausal | 30 | 85 | 166 | 9.12 |
| 04314003 | 70 | Postmenopausal | 80 | 3 | 3 | 9.45 |
| 07100488 | 66 | Postmenopausal | 50 | 7 | 11 | 5.88 |
| 07566314 | 57 | Postmenopausal | 70 | 3 | 3 | 12.23 |
| 09150039 | 60 | Postmenopausal | 60 | 0 | 0 | 7.95 |
| 09269592 | 73 | Postmenopausal | 80 | 70 | 135 | 11.46 |
| 09458744 | 65 | Postmenopausal | 70 | 75 | 175 | 14.53 |
| 09599315 | 62 | Postmenopausal | 40 | 98 | 190 | 11.84 |
| 14480786 | 68 | Postmenopausal | 95 | 98 | 248 | 10.62 |
| 15070758 | 59 | Postmenopausal | 95 | 98 | 200 | 14.44 |
| 23151466 | 67 | Postmenopausal | 60 | 85 | 138 | 17.12 |
| 23698483 | 63 | Postmenopausal | 30 | 100 | 278 | 10.40 |
| 23811739 | 80 | Postmenopausal | 90 | 100 | 273 | 27.94 |
| 27162504 | 42 | Premenopausal | 50 | 97 | 196 | 14.82 |
| 32208416 | 55 | Perimenopausal | 90 | 90 | 195 | 0.22 |
| 42999755 | 60 | Postmenopausal | 40 | 85 | 140 | 11.30 |
| 46813066 | 72 | Postmenopausal | 70 | 100 | 250 | 4.36 |
| 47365415 | 63 | Postmenopausal | 90 | 98 | 255 | 9.23 |
| 55370021 | 49 | Unknown | 80 | 83 | 170 | 11.67 |
| 56304671 | 31 | Premenopausal | 70 | 93 | 163 | 19.69 |
| 64887591 | 65 | Postmenopausal | 80 | 70 | 120 | 8.83 |
| 66438258 | 64 | Postmenopausal | 60 | 83 | 173 | 10.44 |
| 69046268 | 69 | Postmenopausal | 90 | 100 | 258 | 4.80 |
| 69517641 | 45 | Premenopausal | 80 | 98 | 250 | 21.37 |
| 78415558 | 43 | Premenopausal | 40 | 95 | 190 | 22.19 |
| 83849892 | 71 | Postmenopausal | 95 | 97 | 194 | 15.07 |
| 92242302 | 58 | Postmenopausal | 80 | 98 | 248 | 9.34 |
| 93070915 | 85 | Postmenopausal | 95 | 1 | 1 | 1.33 |
| 99070404 | 63 | Postmenopausal | 95 | 38 | 55 | 23.00 |

Abbreviation: ER: estrogen receptor, HGSC: high-grade serous ovarian carcinoma

* Scores were assessed by two independent expert gynecological pathologists (SLB and MHFML-B) in annotated HGSC areas. Mean ER protein expression and ER histoscores are described.
